# Silencing USP19 alleviates cigarette smoke extract-induced mitochondrial dysfunction in BEAS-2B cells by targeting FUNDC1

**DOI:** 10.1515/med-2023-0798

**Published:** 2023-10-06

**Authors:** Yanjing You, Huijuan Wang, Qing Wang, Zongyang Yu, Zhongquan Zhao, Liying Zhuang, Shengyuan Zeng, Jinyang Zheng, Wen Wen

**Affiliations:** Department of Respiratory and Critical Care Medicine, Fuzhou General Hospital of Fujian Medical University, Dongfang Hospital of Xiamen University, 900TH Hospital of Joint Logistics Support Force, PLA, Fuzhou 350025, Fujian, P.R. China; Graduate College of Fujian Medical University, Fuzhou 350025, China; Department of Respiratory and Critical Care Medicine, The Third Affiliated People’s Hospital of Fujian University of Traditional Chinese Medicine, Fuzhou 350108, Fujian, China; Department of Respiratory and Critical Care Medicine, Fuzhou General Hospital of Fujian Medical University, Dongfang Hospital of Xiamen University, 900TH Hospital of Joint Logistics Support Force, PLA, No. 156, Xi’erhuan North Road, Gulou District, Fuzhou 350025, Fujian, P.R. China

**Keywords:** chronic obstructive pulmonary disease, FUNDC1, USP19, mitochondrial function, cigarette smoke extract

## Abstract

Chronic obstructive pulmonary disease (COPD) is commonly caused by smoking. FUN14 domain-containing protein 1 (FUNDC1) plays a fundamental role in mitochondrial autophagy and apoptosis in cigarette smoke extract (CSE)-treated BEAS-2B cells. The present study investigated the mechanism of action of FUNDC1 in mitochondrial dysfunction and apoptosis in CSE-treated BEAS-2B cells. The interaction between ubiquitin-specific peptidase 19 (USP19) and FUNDC1 was analyzed using co-immunoprecipitation. Effects of USP19 knockdown and/or FUNDC1 overexpression on the survival, apoptosis, mitochondrial membrane potential, and oxygen consumption rate (OCR) of BEAS-2B cells treated with 15% CSE were determined. In BEAS-2B cells, CSE inhibited cell survival, promoted apoptosis, increased the expression of USP19 and FUNDC1, increased the ratio of LC3 II to LC3 I (LC3 II/I), and decreased mitochondrial membrane potential and TOM20 levels. In CSE-treated BEAS-2B cells, USP19 knockdown reduced FUNDC1 and LC3 II/I, increased the levels of TOM20, improved cell survival, mitochondrial membrane potential, and OCR, and inhibited apoptosis. USP19 deubiquitinates FUNDC1. FUNDC1 overexpression inhibited the effect of USP19 knockdown in CSE-treated BEAS-2B cells. Overall, decreasing USP19 expression alleviates CSE-induced mitochondrial dysfunction in BEAS-2B cells by downregulating FUNDC1, providing novel insights into the molecular mechanism of FUNDC1 regulation in COPD.

## 1 Introduction

Chronic obstructive pulmonary disease (COPD) is a leading cause of death worldwide. COPD is a lung disease clinically characterized by chronic respiratory symptoms and airflow limitation associated with airway and/or alveolar damage [1,2]. COPD is caused by prolonged exposure to harmful particles or gases, especially tobacco smoke, and accounts for nearly 90% of cases [3,4]. Therefore, studying the mechanism of action of cigarette smoke extract (CSE) on cells of the respiratory system can contribute to the development of new drugs for COPD.

In patients with COPD, mitochondrial morphological abnormalities are observed, including swelling, elongation, fragmentation, and loss of cristae, leading to mitochondrial dysfunction, such as disruption of the electron transport chain [5,6]. This damage activates the production of excessive reactive oxygen species (ROS) in mitochondria, which are the main ROS producers in cells, and results in oxidative stress [7]. Excessive mitochondrial ROS stimulates inflammatory responses that impair mitophagy [8]. Therefore, investigating the effect of CSE on the mitochondrial function of respiratory system cells advances our understanding of the mechanism of smoking-induced damage to the respiratory system.

Mitophagy is highly associated with the ubiquitin-proteasome system and the autophagy-lysosomal pathway [9,10]. The former is the most well-known mechanism of mitophagy, which is mediated by parkin and phosphatase and tensin homologue (PTEN) and the PTEN-induced putative kinase 1 pathway [11,12]. The latter is mediated by mitophagy receptors localized on the outer membrane that recruit autophagosomes to degrade dysfunctional mitochondria [13]. Recently, FUN14 domain-containing protein 1 (FUNDC1) was identified, and has been confirmed to be a novel mitophagy receptor [14,15]. FUNDC1 expression is upregulated in COPD, and FUNDC1 silencing suppresses mitophagy and alleviates apoptosis in bronchial epithelial cells under hypoxic conditions [16]. These findings indicate that FUNDC1 is a potential therapeutic target. However, the mechanism underlying FUNDC1 regulation in COPD remains unknown. Under hypoxic stress, ubiquitin-specific peptidase (USP) 19 binds to and stabilizes FUNDC1, which is involved in the regulation of mitochondrial dynamics [17,18]. Skeletal muscle atrophy caused by smoking is associated with the upregulation of USP19 [19]. USP19 may interact with FUNDC1, which regulates mitophagy in COPD. However, whether USP19 targets FUNDC1 and functions in the respiratory cells of patients with COPD remains unknown.

In the present study, we investigated the role of USP19 in the regulation of FUNDC1, which is involved in mitophagy and apoptosis, in CSE-treated BEAS-2B cells.

## 2 Materials and methods

### 2.1 Cell culture

The human bronchial epithelial cell line (BEAS-2B) was purchased from Xiamen Immocell Biotechnology Co., Ltd (Xiamen, China). BEAS-2B cells were cultured in Roswell Park Memorial Institute (RPMI)-1640 medium (Gibco, NY, USA) supplemented with 10% fetal bovine serum, 100 U/mL penicillin, and 100 U/mL streptomycin. BEAS-2B cells were incubated at 37°C with 5% CO_2_ concentration.

### 2.2 Treatment of CSE

To induce COPD *in vitro*, CSE was added to the BEAS-2B cell culture medium. CSE was prepared using a modified syringe-driven apparatus in which one cigarette was combusted, and smoke was bubbled through 5 mL of RPMI-1640 medium to obtain a 100% CSE solution [20]. The 100% CSE solution subsequently was filtered through an aseptic 0.22 μm filter, and diluted into 30, 25, 20, 15, and 10% CSE solution in medium for the culture of BEAS-2B cells. After 24 h, the 2,5-diphenyl-2*H*-tetrazolium bromide (MTT) assay was used to detect cell survival. A CSE solution that decreased BEAS-2B cells survival by 50% was selected to induce COPD in the present study.

### 2.3 Grouping of cells

Plasmids encoding FUNDC1 (FUNDC1 OE or Flag-FUNDC1), USP19 (HA-USP19), or short hairpin RNA (shRNA) targeting USP19 (shUSP19-1, -2, and -3) and the corresponding negative control plasmids (shNC and vector) were purchased from XIAMEN Anti-HeLa Biological Technology Trade Co. Ltd (Xiamen, China). BEAS-2B cells were divided into shNC, shNC + 15% CSE, shUSP19 + 15% CSE, and shUSP19 + FUNDC1 OE + 15% CSE groups. After cell transfection of 5 μg of plasmid (2.5 μg of shUSP19 or shNC and 2.5 μg of FUNDC1 OE or vector) in a six-well plate for 16 h, the supernatant of the cells was replaced with medium containing 15% CSE. The cells were cultured for 24 h and collected for subsequent detection. All primers used for plasmid construction are listed in Table 1.

**Table 1:** Primers used in this study

Primers		Sequence (5′–3′)
ShUSP19-1	F	CCGGCTCCACTGCGAGCGAAGTATTCTCGAGAATACTTCGCTCGCAGTGGAGTTTTT
	R	AATTAAAAACTCCACTGCGAGCGAAGTATTCTCGAGAATACTTCGCTCGCAGTGGAG
ShUSP19 -2	F	CCGGCCGGTACTCTGTGAGTGTATTCTCGAGAATACACTCACAGAGTACCGGTTTTT
	R	AATTAAAAACCGGTACTCTGTGAGTGTATTCTCGAGAATACACTCACAGAGTACCGG
ShUSP19 -3	F	CCGGGCTGCCCAGCTACGATCTATACTCGAGTATAGATCGTAGCTGGGCAGCTTTTT
	R	AATTAAAAAGCTGCCCAGCTACGATCTATACTCGAGTATAGATCGTAGCTGGGCAGC
FUNDC1 OE	F	TAGAGAATTCGGATCCATGGCGACCCGGAACCCCCC
	R1	TGTCGTCATCGTCTTTGTAGTCAGATGCAAGTCCGAGCAAAAAGCCTCCC
	R2	GCTTCCATGGCTCGAGTCACTTGTCGTCATCGTCTTTGTAG
USP19 OE	F	CTAGAGAATTCGGATCCATGTCTGGCGGGGCCAGTG
	R	AGCTTCCATGGCTCGAGTCATCTCCAGCGACTCTGGG
18S	QF	AGGCGCGCAAATTACCCAATCC
	QR	GCCCTCCAATTGTTCCTCGTTAAG
USP19	QF	GCTGCTATCCTCAGAGTTGGCT
	QR	TCATCCTCCGACTGTTGCTTCC
FUNDC1	QF	ATTGAAGAAGCAACAGAA
	QR	ATAGTTGAATCCGTTATGG

Notes: F: forward primer for plasmid, R: reverse primer for plasmid, QF: forward primer for qPCR, QR: reverse primer for qPCR.

### 2.4 Cell survival assay

BEAS-2B cells at 24 h post-treatment were seeded into 96-well plates (10^4^ cells per well). Cells were harvested and incubated with MTT solution (Beyotime Biotechnology, Shanghai, China). Cell survival was determined by measuring the optical density using an absorbance reader (Bio-Tek, Winooski, USA) at 490 nm.

### 2.5 Apoptosis assay

Harvested BEAS-2B cells were stained with annexin V-fluorescein isothiocyanate (Vazyme, Nanjing, China) and propidium iodide (Vazyme). The apoptosis rate was determined based on the fluorescence signals using a flow cytometer (BD Biosciences, Franklin Lakes, NJ, USA).

### 2.6 Mitochondrial membrane potential assay

Harvested BEAS-2B cells were stained with 5,5′,6,6′-tetrachloro-1,1′,3,3′-tetraethyl-imidacarbocyanine iodide (JC-1; Invitrogen), according to the manufacturer’s instructions. The mitochondrial membrane potential was estimated based on fluorescence signals using a flow cytometer (BD Biosciences).

### 2.7 Oxygen consumption rate (OCR) measurement

The harvested BEAS-2B cells were transferred to an oxygraph chamber containing respiration buffer. The OCR was determined using a Seahorse XF96 analyzer (Seahorse Bioscience, North Billerica, MA, USA) and an XF Cell Mito Stress Test Kit (Seahorse Bioscience). After measuring OCR at basal respiration, 2.5 μM oligomycin was added to measure residual proton leak, 1 μM carbonyl cyanide-4-(trifluoromethoxy) phenylhydrazone was added to induce maximum respiration, and 2.5 μM antimycin A/rotenone was added for adenosine triphosphate (ATP) production.

### 2.8 Quantitative PCR (qPCR)

Total RNA was isolated from BEAS-2B cells using TRIzol reagent (Takara, Dalian, China). The quality of total RNA was verified using gel electrophoresis, and a Nanodrop 2000 spectrometer was used to determine RNA concentration (Thermo Fisher Scientific, USA). A reverse transcription kit (Applied Biosystems, Foster City, CA, USA) was used to obtain cDNA for qPCR. qPCR was performed on a QuantStudio 5 system (Applied Biosystems, USA) using the SYBR Green Master Mix (Thermo Fisher Scientific, USA). The 2^−ΔΔCT^ algorithm was used to determine the relative expression of mRNA. Information on the qPCR primers is provided in Table 1.

### 2.9 Western blotting

BEAS-2B cells were lysed in radioimmunoprecipitation assay buffer supplemented with protease and phosphatase inhibitors (Beyotime, Shanghai, China). Cell lysates were separated using sodium dodecyl sulfate-polyacrylamide gel electrophoresis and subsequently transferred to a polyvinylidene difluoride membrane. The membrane was treated with primary antibodies after blocking with 5% skim milk, and then with secondary antibodies that had been conjugated to horseradish peroxidase (HRP). Protein levels were estimated based on protein intensity. The protein intensity was determined using a film processing system (iBright FL1000; Thermo Fisher Scientific) with an HRP chemiluminescence kit (Tiangen Biotech, Beijing, China). The antibodies used for western blotting are listed in Table 2.

**Table 2:** Antibodies for western blotting

Antibodies	Manufacturer	Catalog no.	Dilution
Actin	Proteintech, Wuhan, China	81115-1-RR	1:5,000
USP19	Proteintech, Wuhan, China	25768-1-AP	1:3,000
FUNDC1	Abcam, Shanghai, China	ab224722	1:2,000
TOM20	Proteintech, Wuhan, China	11802-1-AP	1:3,000
LC3 I/II	Proteintech, Wuhan, China	14600-1-AP	1:3,000
DYKDDDDK Tag	Proteintech, Wuhan, China	80010-1-RR	1:4,000
HA-tag	Proteintech, Wuhan, China	51064-2-AP	1:4,000
His-tag	Proteintech, Wuhan, China	10001-0-AP	1:4,000
HRP goat anti-rabbit IgG (H + L)	Proteintech, Wuhan, China	SA00001-2	1:10,000

### 2.10 Co-immunoprecipitation (co-IP)

Plasmids HA-USP19 and Flag-FUNDC1 were co-transfected into BEAS-2B cells to elucidate the interaction between USP19 and FUNDC1. To understand the deubiquitination of FUNDC1 by USP19, shUSP19 and/or Flag-FUNDC1 expression plasmids were co-transfected with His-Ub expression plasmids into BEAS-2B cells. After 24 h, the BEAS-2B cells were lysed with immunoprecipitation buffer (150 mmol/L NaCl; 50 mmol/L Tris (hydroxymethyl) aminomethane hydrochloride, pH = 7.4; 40 mmol/L glycerophosphate; 1 mmol/L Na_4_OV_3_; 10 mmol/L NaF; and 2 mmol/L ethylenediaminetetraacetic acid) supplemented with 1 mmol/L phenylmethylsulphonyl fluoride and a protease inhibitor. Cell lysates were incubated with DYKDDDDK tag antibody (catalog number: 66008-4-Ig; Proteintech) or HA tag antibody (catalog number: 66006-2-Ig; Proteintech) overnight, followed by incubation with Protein A/G beads. Immunoprecipitates were subjected to western blotting, followed by washing with immunoprecipitation buffer.

### 2.11 Analysis of USP19-stabilizing FUNDC1 protein

To understand the effect of USP19 on FUNDC1 protein level, shUSP19 and Flag-FUNDC1 expression plasmid co-transfected cells were treated with 50 μg/mL cycloheximide. After 0, 2, 4, 8, and 12 h, cells were harvested for western blotting.

### 2.12 Statistical analysis

All statistical analyses were conducted using GraphPad Prism (version 5.0; San Diego, California, USA). Student’s *t*-test was used to compare the differences between two groups, the student’s *t* test was utilized. A one-way analysis of variance was used to compare multiple groups. *P* < 0.05 indicated a significant difference. Prior to the parameter tests, the normality of the data was verified using the Shapiro–Wilk test.

## 3 Results

### 3.1 CSE inhibits cell survival, enhances cell apoptosis, decreases mitochondrial membrane potential, and increases the expression of USP19 and FUNDC1

**Figure 1:**
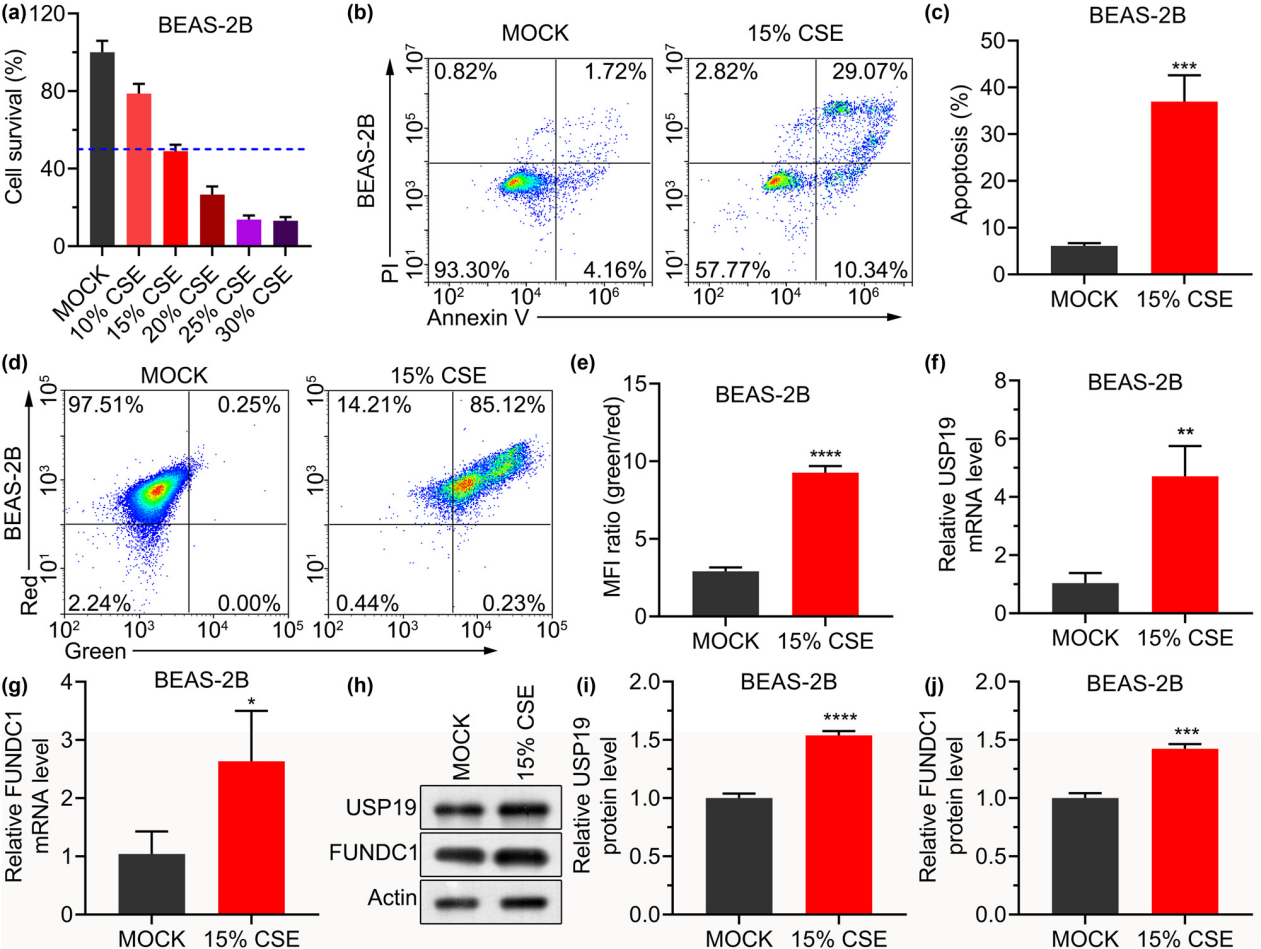
CSE inhibits cell survival, enhances cell apoptosis, decreases mitochondrial membrane potential, and increases the expression of USP19 and FUNDC1. (a) Survival of BEAS-2B cells treated with 10, 15, 20, 25 and 30% CSE. (b) Effect of 15% CSE treatment on the apoptosis of BEAS-2B cells. (c) Apoptosis rates of BEAS-2B cells in each group. (d) Effect of 15% CSE on the mitochondrial membrane potential of BEAS-2B cells. (e) MFI of each group. (f)–(j) mRNA (f) and (g) and protein (h)–(j) levels of USP19 and FUNDC1 in BEAS-2B cells treated with 15% CSE. Data are expressed as mean ± standard deviation (SD) (a), (c), (e)–(j). *, **, ***, **** indicated *P* <0.05, <0.01, <0.001, and <0.0001, respectively.

To construct a COPD cell model, BEAS-2B cells were stimulated with CSE. CSE treatment showed that the survival of BEAS-2B cells was decreased by CSE (Figure 1a). The decrease was positively correlated with the CSE concentration from 10 to 30%, and the median inhibitory concentration (IC50) was 15%. The CSE solution (15%) increased the percentage of apoptotic cells (Figure 1b and c). CSE of 15% also significantly increased the mean fluorescence intensity (MFI) ratio of green to red in BEAS-2B cells, indicating that CSE reduced the mitochondrial membrane potential in cells (Figure 1d and e). Moreover, studies have shown that FUNDC1 is involved in COPD progression and is stabilized by USP19 [16,17]. Therefore, we examined the expression of USP19 and FUNDC1 in CSE-treated cells. The results showed that 15% CSE significantly increased the relative mRNA and protein expression levels of USP19 and FUNDC1 in BEAS-2B cells (Figure 1f–j). These results revealed that 15% CSE can be used to construct a cell model of COPD and that 15% CSE increases USP19 and FUNDC1 expression.

### 3.2 USP19 is involved in apoptosis and mitochondrial dysfunction in BEAS-2B cells stimulated with CSE

**Figure 2:**
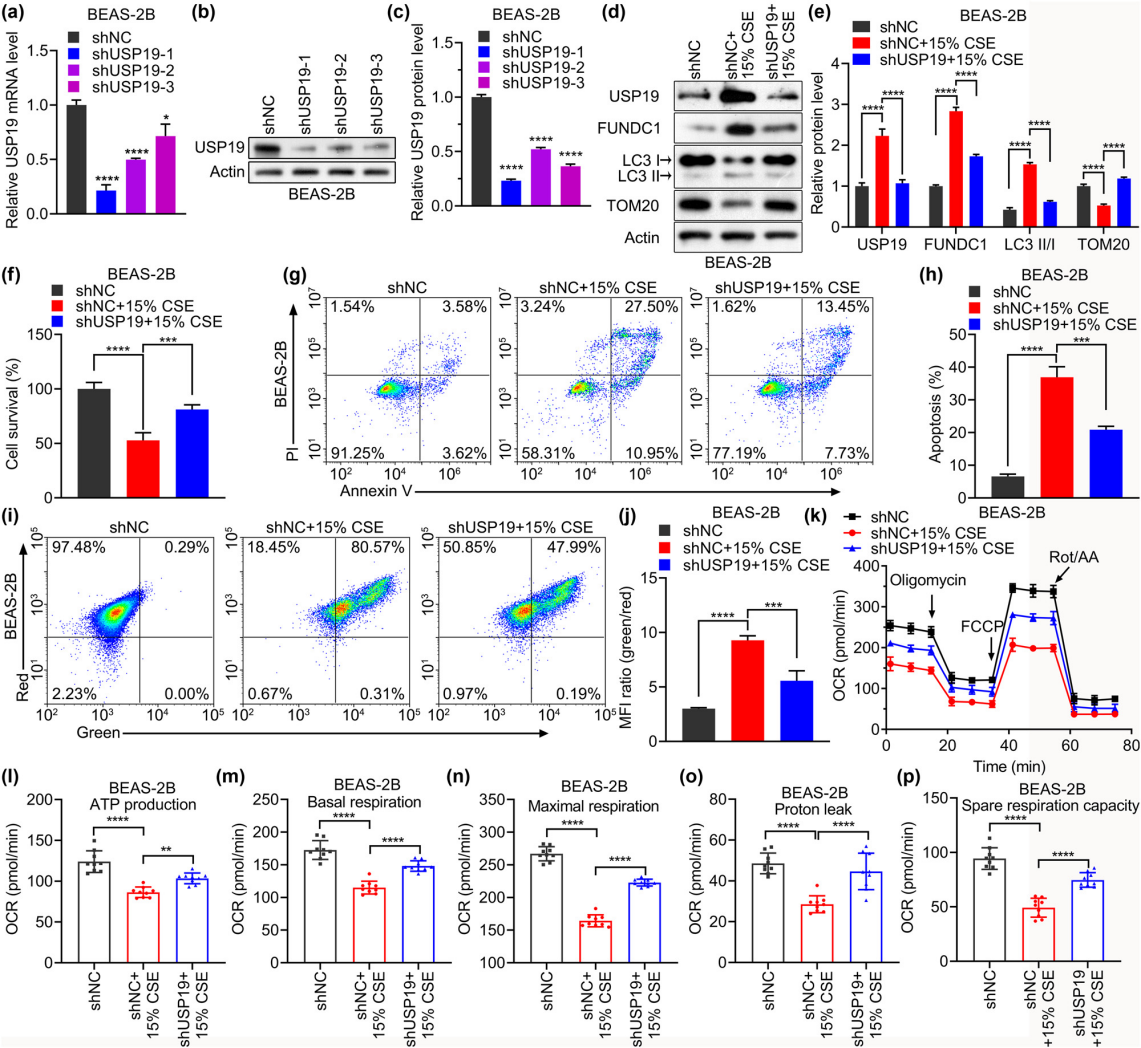
USP19 is involved in apoptosis and mitochondrial dysfunction in BEAS-2B cells stimulated with CSE. (a)–(c) Knockdown efficiency of USP19 shRNA (shUSP19-1, -2, and -3) at the mRNA (a) and protein (b) and (c) levels. (d)–(p) Effects of shUSP19 and CSE treatment on the protein levels of FUNDC1, LC3 II/I, and TOM20 (d) and (e), cell survival (f), cell apoptosis (g) and (h), mitochondrial membrane potential (i) and (j), and OCR (k)–(p) in BEAS-2B cells. Ns: not significantly different. Data are expressed as the mean ± SD (a), (c), (e), (f), (h), (j), (k)–(p). *, **, ***, and **** denoted *P* <0.05, <0.01, <0.001, and <0.0001, respectively.

To further explore the role of USP19 in COPD development, we knocked down USP19 in CSE-stimulated BEAS-2B cells. The USP19 mRNA and protein expression levels were significantly decreased by shUSP19-1, -2, and -3 in BEAS-2B cells (Figure 2a–c). Among these, shUSP19-1 was the most efficient for USP19 knockdown; therefore, the following experiments were carried out using shUSP19-1. USP19 and FUNDC1 protein levels and the ratio of LC3 II to LC3 I (LC3 II/I) were lower and TOM20 protein expression was higher in the shUSP19 + 15% CSE group than in the shNC + 15% CSE group (Figure 2d and e). shUSP19 significantly improved cell survival (Figure 2f), suppressed apoptosis (Figure 2g and h), and alleviated mitochondrial membrane potential reduction (Figure 2i and j) in CSE-stimulated BEAS-2B cells. Moreover, CSE significantly reduced the OCR in BEAS-2B cells, whereas shUSP19 mitigated this effect (Figure 2k). In BEAS-2B cells treated with CSE, OCR was significantly increased by shUSP19 in terms of ATP production, basal respiration, maximal respiration, proton leakage, and spare respiration capacity (Figure 2l–p). These findings implied that USP19 knockdown alleviated CSE-induced apoptosis and impaired mitochondrial function.

### 3.3 USP19 stabilized FUNDC1 in BEAS-2B cells

**Figure 3:**
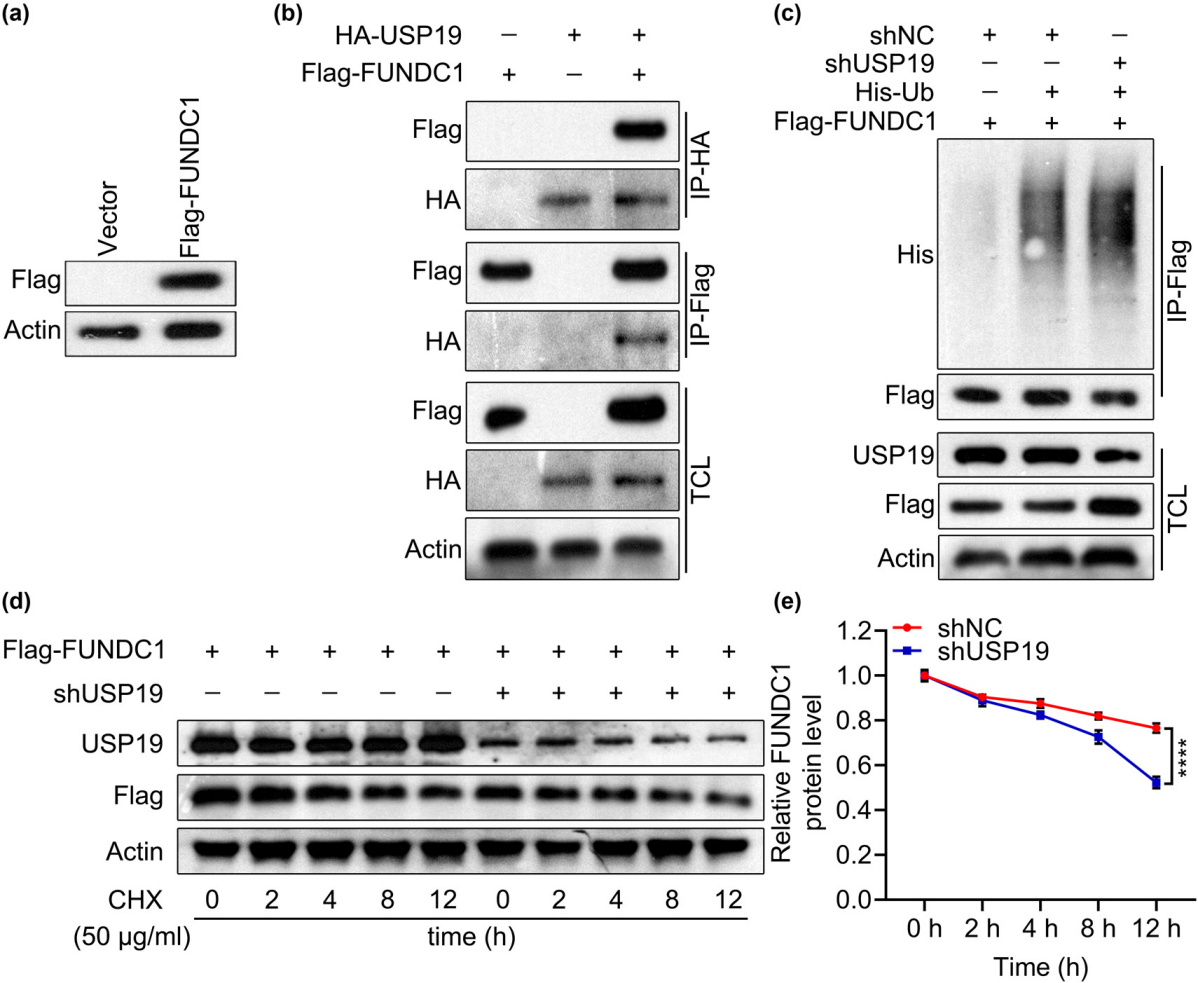
USP19 stabilized FUNDC1 through deubiquitination. (a) Detection of Flag-FUNDC1 protein expressed by the plasmid flag-FUNDC1. (b) Interaction between USP19 and FUNDC1 was determined by co-IP assay. (c) FUNDC1 ubiquitination was measured using co-IP assay after depletion of USP19 in BEAS-2B cells. (d) and (e) FUNDC1 degradation is detected after USP19 expression is reduced in BEAS-2B cells. Data are expressed as the mean ± SD (e). **** denoted *P* < 0.0001.

A previous study demonstrated that USP19 interacts with FUNDC1 and stabilizes FUNDC1 [17]. In the present study, we verified this using exogenous co-IP. Flag-FUNDC1 was successfully expressed in BEAS-2B cells transfected with Flag-FUNDC1 plasmid (Figure 3a). In co-transfected BEAS-2B cells, FUNDC1 was pulled down by USP19 using an anti-HA antibody and USP19 was pulled down by FUNDC1 using an anti-Flag antibody (Figure 3b). These results indicated that FUNDC1 interacts with USP19. In BEAS-2B cells co-transfected with FUNDC1, shUSP19, and ubiquitin, ubiquitin was pulled down by FUNDC1 using an anti-FLAG antibody and the protein levels of both FUNDC1 and ubiquitin were increased by shUSP19 (Figure 3c). FUNDC1 protein levels decreased from 0 to 12 h after cycloheximide treatment, which was significantly accelerated by shUSP19 in BEAS-2B cells (Figure 3d and e). Collectively, these results showed that USP19 interacts with FUNDC1 and stabilizes FUNDC1 in BEAS-2B cells.

### 3.4 USP19 regulates apoptosis and mitochondrial dysfunction through FUNDC1 in BEAS-2B cells stimulated with CSE

**Figure 4:**
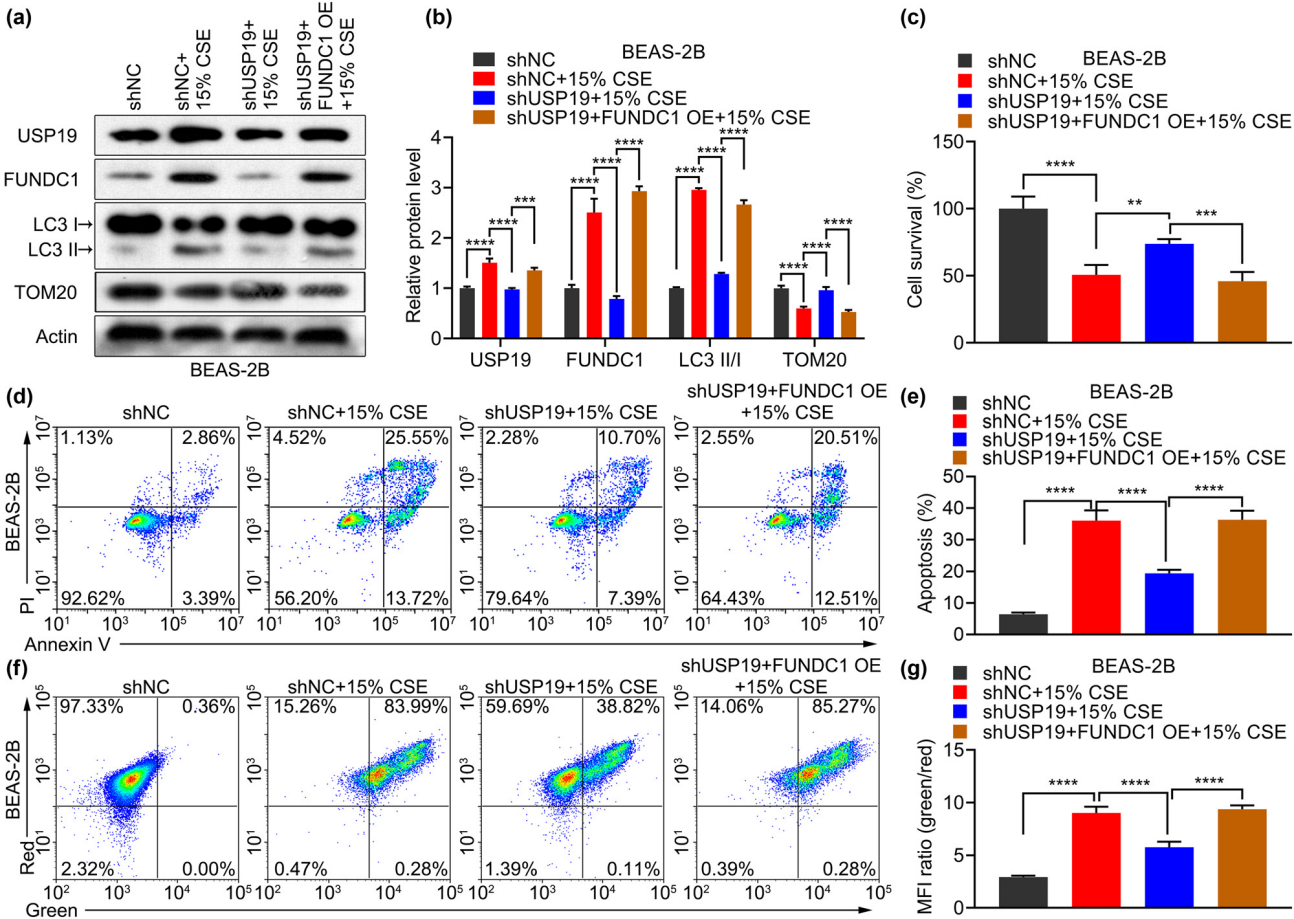
USP19 regulate apoptosis and mitochondrial membrane potential through FUNDC1 in BEAS-2B cells stimulated with CSE. (a)–(g) After CSE-treated BEAS-2B cells were transfected with plasmids shUSP19 and FUNDC1 OE, protein levels of FUNDC1, LC3 II/I, TOM20 (a) and (b), cell survival (c), cell apoptosis (d) and (e), and mitochondrial membrane potential (f) and (g) were detected. Data are expressed as the mean ± SD (b), (c), (e), and (g). *P* <0.05, <0.01, <0.001, and <0.0001 are represented by *, **, ****, and ****, respectively. Ns: not significantly different.

**Figure 5:**
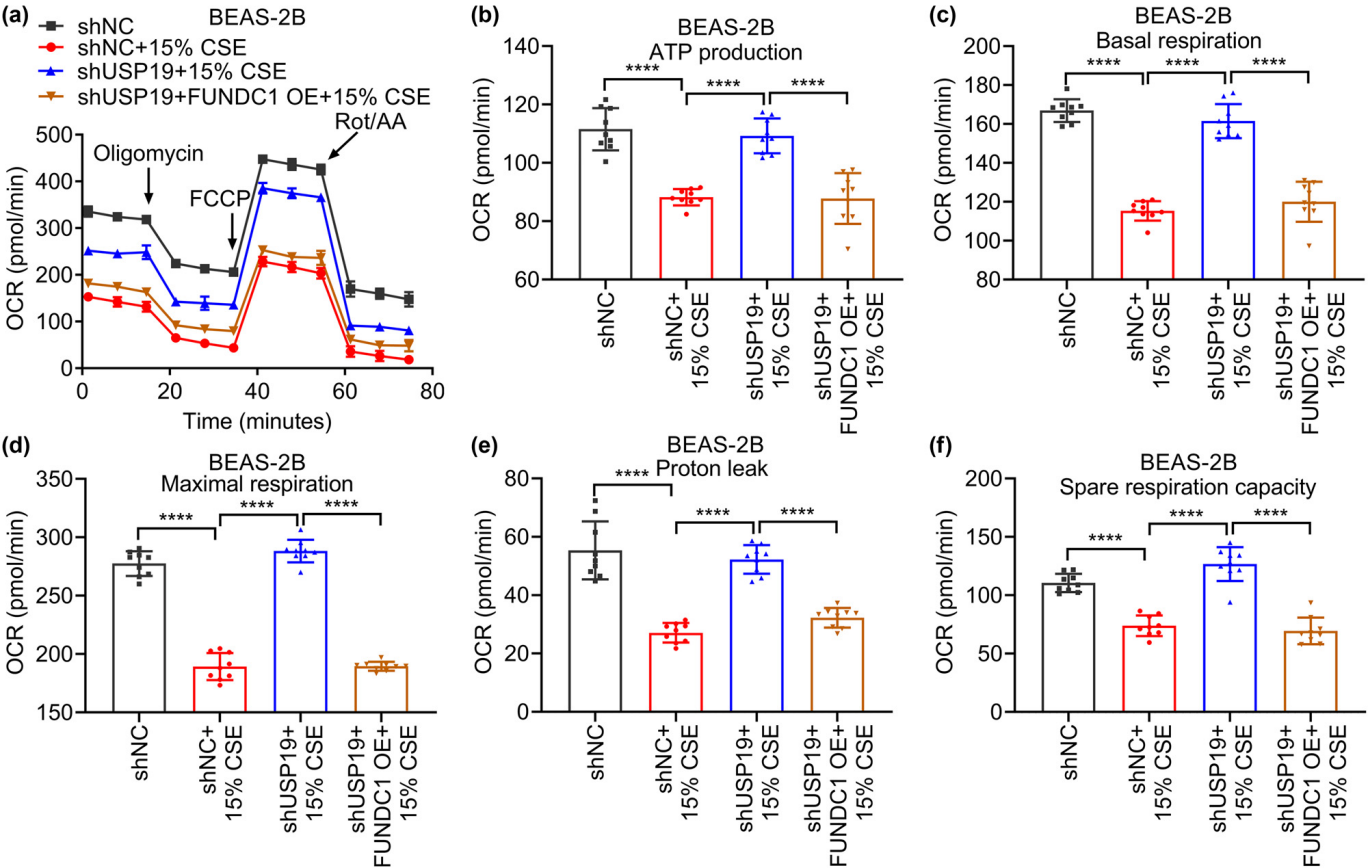
USP19 is involved in mitochondrial OCR through FUNDC1. (a)–(f) Effects of USP19 knockdown and/or FUNDC1 expression on cell OCR (a) and OCR for ATP production (b), basal respiration (c), maximal respiration (d), proton leak (e), and spare respiration capacity (f) of CSE-treated BEAS-2B cells. Data are expressed as mean ± SD (a)–(f). *P* <0.05, <0.01, <0.001, and <0.0001 are represented by *, **, ***, and ****, respectively. Ns: not significantly different.

To investigate whether USP19 is involved in the development and progression of COPD through FUNDC1, we overexpressed FUNDC1 while knocking down USP19 in CSE-treated BEAS-2B cells. FUNDC1 overexpression significantly increased the protein levels of USP19, FUNDC1, and LC3 II/I, and decreased TOM20 protein level in BEAS-2B cells co-transfected with shUSP19 and FUNDC1 OE and treated with CSE (Figure 4a and b). Moreover, FUNDC1 overexpression significantly alleviated the effects of shUSP19 on cell survival (Figure 4c), apoptosis (Figure 4d and e), and mitochondrial expression (Figure 4f and g) in CSE-treated BEAS-2B cells. Additionally, FUNDC1 overexpression significantly alleviated the effects of shUSP19 on OCR during basal respiration, maximal respiration, proton leakage, spare respiration, and ATP production in CSE-treated BEAS-2B cells (Figure 5a–f). USP19 promotes CSE-induced apoptosis and impairs mitochondrial function by stabilizing FUNDC1.

## 4 Discussion

The significant upregulation of FUNDC1 expression in CSE-treated BEAS-2B cells is consistent with previous findings [14,15], confirming that FUNDC1 is involved in the regulation of mitophagy in COPD. In agreement with previous findings [17,18], the mRNA and protein levels of USP19 were also stimulated in these cells. A pattern similar to that of FUNDC1 implied that USP19 may be involved in the regulation of mitophagy in COPD. USP19 localizes to the endoplasmic reticulum membrane and plays a critical role in the endoplasmic reticulum protein degradation (ERAD) pathway [21]. The ubiquitin-proteasome system is involved in the regulation of mitochondrial function [22,23]. USP19 is a crucial regulator of mitophagy in HEK293T cells [24]. USP19 knockdown in CSE-treated BEAS-2B cells increased cell survival, OCR, and protein levels of LC3 II/I, reduced TOM20 protein level, and mitigated the reduction in mitochondrial membrane potential. These findings indicated that USP19 plays an important role in the regulation of mitochondrial function in patients with COPD. Specifically, USP19 removes the K11 linked ubiquitin chains that stabilizes beclin1 and promotes mitophagy. In the present study, FUNDC1 protein levels were decreased by USP19 knockdown, indicating that USP19 may be involved in mitophagy by regulating FUNDC1 expression in CSE-treated BEAS-2B cells.

The interaction between USP19 and FUNDC1 was confirmed using the co-IP assays. The degradation of FUNDC1 was increased by USP19 knockdown, indicating that USP19 contributes to FUNDC1 stabilization. USP19, a deubiquitination enzyme in the ERAD pathway, prevents protein degradation through the proteasome [21], indicating that USP19 stabilizes FUNDC1 through deubiquitination. Moreover, the increased protein levels of LC3 II/I and cell survival induced by USP19 knockdown were alleviated by FUNDC1 overexpression in CSE-treated BEAS-2B cells. In addition, the effects of USP19 knockdown on MMP and OCR were eliminated by FUNDC1 overexpression in CSE-treated BEAS-2B cells. FUNDC1 is a positive regulator of mitophagy COPD [16]. These findings indicate that USP19 promotes mitophagy by stabilizing FUNDC1 in CSE-treated BEAS-2B cells. In addition to FUNDC1, USP19 may regulate the stabilization of beclin1, leading to increased mitophagy in COPD [24]. Further investigations are required to explore these regulatory effects in COPD.

In the present study, apoptosis was stimulated by CSE in BEAS-2B cells, which is consistent with previous findings [25]. The rate of stimulated apoptosis was alleviated by USP19 knockdown, which is consistent with mitophagy. A similar pattern implies that mitophagy and apoptosis interact during COPD. In general, mitophagy and autophagy decrease oxidative stress and maintain cell survival, leading to the suppression of apoptosis. However, excessive mitophagy and autophagy promotes apoptosis [13]. A previous report has confirmed that autophagy is involved in apoptosis regulation in CSE-treated BEAS-2B cells [26]. Macroautophagy promotes apoptosis in BEAS2-B cells, whereas chaperone-mediated autophagy inhibits apoptosis. Therefore, the interplay between mitophagy and autophagy requires further investigation in patients with COPD.

COPD is the fourth leading cause of death worldwide. Although numerous therapeutic approaches and management strategies have been developed, COPD appears to be difficult to reverse and patients are at high risk of exacerbation [4,27]. Our findings revealed that USP19 is a positive regulator of FUNDC1, which is involved in mitochondrial dysfunction and apoptosis in CSE-treated BEAS-2B cells through deubiquitination. The knockdown of USP19 alleviated mitochondrial dysfunction and apoptosis in CSE-treated BEAS-2B cells. These findings suggest that USP19 is a potential therapeutic target for COPD. However, the clinical effects and value of targeting USP19 remain to be investigated in patients in the further study.
